# Smoking and Fibrocystic Changes in the Breast: A Case Report of a Lifelong Smoker and Changes in Breast Parenchyma

**DOI:** 10.7759/cureus.26384

**Published:** 2022-06-28

**Authors:** Dujanah S Bhatti, Muhammad Haseeb T Bokhari, Muhammad Adil A Khan

**Affiliations:** 1 Surgery, Aberdeen Royal Infirmary, Aberdeen, GBR; 2 Surgery, Shaikh Zayed Medical Complex, Lahore, PAK; 3 Plastic Surgery, Aberdeen Royal Infirmary, Aberdeen, GBR

**Keywords:** bleeding risk, modified radical mastectomy (mrm), stromal fibrosis, cigarette smoking, breast cancer

## Abstract

Smoking is a well-known risk factor for breast cancer, and the nicotine in cigarette smoke has been associated with fibrotic changes in the breast. Although considered benign, these changes have the potential to make surgical dissection more difficult and may increase the risk of surgical complications. Here we discuss the case of a middle-aged female who was a lifelong smoker with breast cancer and who underwent a simple mastectomy. Intra-operatively, the overall breast parenchyma appeared quite firm, making tissue dissection challenging and the operating surgeon had to endure to remove the tumor and the remaining breast tissue.

## Introduction

The effects of smoking on the overall health of an individual can be multifocal [1]. These changes stem from the harmful chemical in cigarettes. Continuous exposure to these chemicals alters the natural progression of growth, architectural layout, and differentiation of the cells [1,2]. We observed these changes in breast parenchyma in our study, in which we surgically dissected and removed a breast tissue to find the changes in its parenchyma and texture.

The role and influence of cigarette smoking on the breast can range from small epithelial fibrocystic changes (that can be benign) to more aggressive and deathly conditions like breast cancer. These changes can be correlated to ionizing radiation from radiotherapy, which produces the same detrimental effects on human tissues [3].

Overall, the hypoxic environment created by cigarette smoking contributes to poor healing, more risk of infectivity, skin changes, and limited options for curative management [2,4]. The duration of smoking has been reported to have a strong correlation with fibrotic changes in the breast. This phenomenon is more strongly associated with the duration of smoking rather than the number of cigarettes per day [5]. In our case, we observe the overall changes in breast parenchyma from 45 years of smoking and the difficulty in surgery. Additionally, we highlight the difficulty in surgery and tissue handling due to this abnormally fibrotic nature of the breast tissue.

According to the National Cancer Institute's (NCI) Surveillance, the incidence of breast cancer is 12.9 percent in a female during her lifetime [6,7]. The relationship between smoking and breast cancer is still unclear; however, the data depicts that this habit has an impact on the risk of developing breast cancer compared to someone who is a non-smoker.

## Case presentation

The patient was a 67-year-old lady with a 45-year history of smoking approximately 45 cigarettes per day and was diagnosed with (left) breast cancer in 2019. She underwent mastectomy (left) and has also completed five years on tamoxifen. She has now presented with a four-day history of a lump in her right breast. The area around the lump was also very painful. With a BMI of 26, upon examination of the right breast, there were no changes to the skin or nipple. There was no visible lump. However, on palpation, we noticed a lump at the 10 o'clock position; it was a pea-sized, soft lump that was more prominent in the sitting position. There was slight tenderness on palpation. She had no axillary nodes. 

Imaging showed a lump, graded M5 on a mammogram, 13 mm, upper outer aspect, right breast - possible fibroadenoma. There was another lump on the same right breast graded as M3, 11 mm in size in the upper inner aspect. Ultrasound showed a right breast upper outer quadrant as U5 and upper inner quadrant as U4.

Ultrasound-guided biopsy was scheduled, which showed invasive carcinoma. Biochemically, the patient had no abnormality to note.

She was scheduled for right breast mastectomy and axillary nodal clearance.

**Figure 1:**
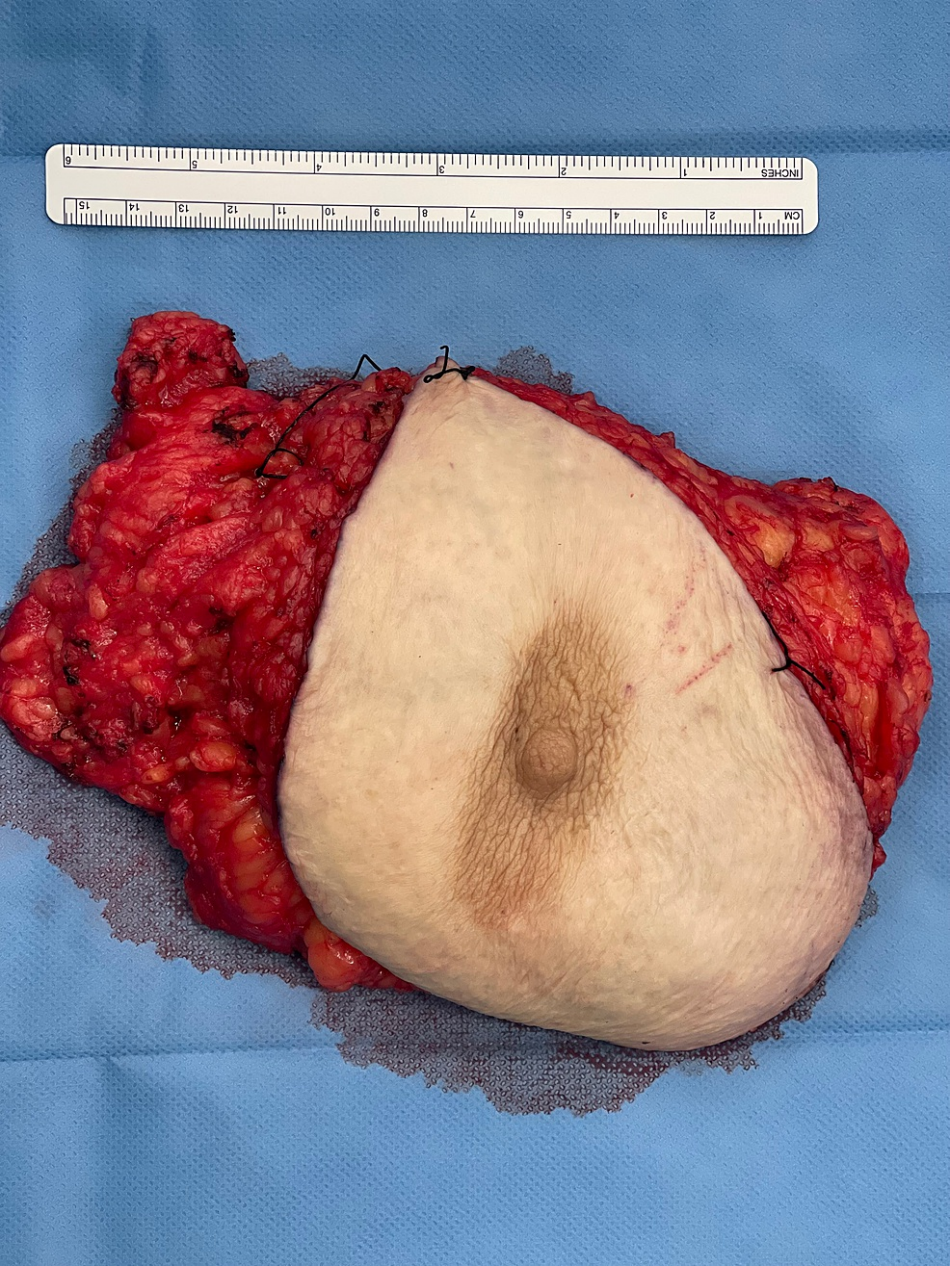
Anterior view of breast tissue showing black sutures to orient The breast tissue has dense, fibrotic fat.

Per operatively, she had slight ptotic breast tissue with palpable tumors on the upper outer and inner quadrant. However, the breast had some features which were not predictable and had a significant alteration to the surgical technique and the use of hemostatic methods. On appearance, the breast tissue (Figure 1), representing the anterior view of the breast tissue, had very dense fat. The breast tissue was firm, and the overall feel of the gland was of a skeletal muscle. It was slightly adherent to the pectoralis facia and had a firm consistency. The weight of the breast tissue was 402 grams.

**Figure 2:**
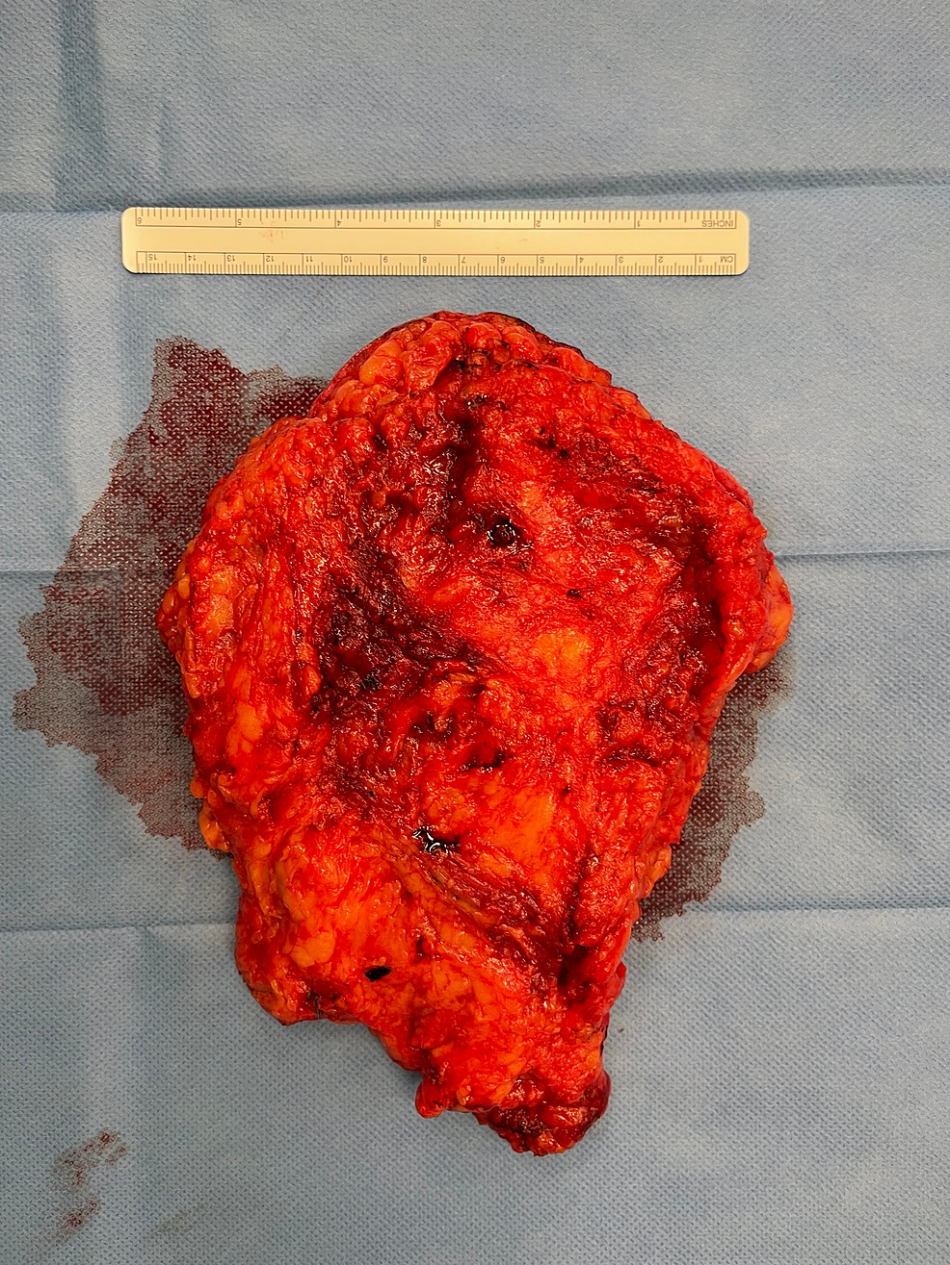
Pectoral aspect of breast tissue showing dark, muscle-like fat

The plane of dissection between the breast tissue and the pectoral fascia was difficult to ascertain, owing to the overall texture and the tight fibrotic nature of the breast parenchyma (Figure 2). Figure 2 represents the posterior (pectoral aspect) of the removed breast tissue. It is bright red and fibrotic and though tissue. This tissue represents the changes that have happened over the course of 45 years due to continuous smoking, and this made the dissection very difficult and the use of ligation clips redundant, as they would either slip or not hold. Larger clips were also tried but in vain. The odd parenchymal architecture of the breast tissue made it challenging to achieve a surgical clearance. However, the breast tissue was meticulously dissected, and elevation was done. The skin was closed, and drains were placed. 

## Discussion

There is a documented increased risk of breast cancer in patients who smoke at least five cigarettes a day or greater than 10 packs per year (a total of 21% increase in breast cancer) [7,8]. This phenomenon can be attributed to the high levels of carcinogens in tobacco contained within a cigarette [9].

Once nicotine is in the bloodstream, it is distributed throughout the body and alters the cellular response to induce fibrosis [10]. The receptors nicotine acts on are called nicotinic acetylcholine receptors (nAChRs), which are located on the cellular members. These receptors are oriented in nature and have a special assembly of subunits that maintain the integrity of the cell and regulate the cellular signaling mechanisms [6,11]. The receptors are modulated by special signals to maintain homeostasis by the cell. Nicotine exerts its effect by binding to these channels and allowing an influx of calcium ions into the cells, which contribute to abnormal and dysregulated growth [6,11,12]. Nicotine can also exert its pathological response to the cells by upregulating the growth of the cells by binding to the alpha subunit of the receptors and resulting in cancers.

Fibrosis can be pathological or physiological in response to the body's own growth or by extrinsic injuries that result in the accumulation of extracellular fibrosis [12]. The origin of fibrosis stems from the abnormal production of collagen or collagen-producing cells. These cellular responses come from fibroblast types of cells that are sensitive to nicotine and are recruited from bone marrow [11,13]. Nicotine promotes the production of fibroblast, and this results in collagen production. Although this pro-fibrotic activity is evident in all organs of the body, we studied the effect of nicotine on the breast in our case. The pathological response to nicotine in our patient significantly affected the breast tissue and impacted the surgery. Although the epidemiological studies advocate that the response of tissue towards nicotine remains non-life threatening, it can potentiate itself to fibrocystic breast changes and ultimately limit the therapeutic options [13].

Our patient was significantly high risk for postoperative surgical site infections owing to her extensive cigarette smoking history. Although she did not develop any surgical site infection (SSI) post-operatively, her past medical history showed a significant number of hospital admission and multiple courses of antibiotics to treat surgical sites in the contralateral breast after mastectomy.

## Conclusions

Breast tissue has a pathological response to nicotine in cigarette smoke. These changes leave the breast tissue in a state of firm fibrotic tissue that hinders dissection and makes surgical clearance difficult for the surgeon. These changes can be prevented by halting smoking and awareness. 
